# Serum Uric Acid Level as a Harbinger of Type 2 Diabetes: A Prospective Observation in Taiwan

**DOI:** 10.3390/ijerph17072277

**Published:** 2020-03-28

**Authors:** Wen-Chih Wu, Yen-Wen Lai, Yu-Ching Chou, Yu-Chan Liao, San-Lin You, Chyi-Huey Bai, Chien-An Sun

**Affiliations:** 1 School of Public Health, National Defense Medical Center, Taipei City 114, Taiwan; doctor0317@yahoo.com.tw (W.-C.W.); trishow@mail.ndmctsgh.edu.tw (Y.-C.C.); sw936229@gmail.com (Y.-C.L.); 2 Department of Surgery, Suao and Yuanshan branches of Taipei Veterans General Hospital, Yilan County 270, Taiwan; 3 Department of Public Health, College of Medicine, Fu-Jen Catholic University, New Taipei City 242, Taiwan; andylai5108@gmail.com; 4 Department of Medicine, College of Medicine, Fu-Jen Catholic University, New Taipei City 242, Taiwan; yousanlin@gmail.com; 5 Big Data Research Center, College of Medicine, Fu-Jen Catholic University, New Taipei City 242, Taiwan; 6 School of Public Health, College of Public Health and Nutrition, Taipei Medical University, Taipei City 110, Taiwan; baich@tmu.edu.tw

**Keywords:** cohort study, type 2 diabetes, uric acid

## Abstract

Background: Current evidence suggests an association of uric acid with diabetes risk, but it is still unclear whether uric acid is merely a risk marker or an independent risk factor. We evaluate the impact of serum uric acid (SUA) levels on the future risk of developing type 2 diabetes, independent of other factors. Methods: A population-based cohort study was conducted among 4130 participants who were found to be free of type 2 diabetes at baseline recruitment in 2002. Baseline SUA measured in 2002 was longitudinally related to the incident type 2 diabetes that occurred during the follow-up period between 2002 and 2007. Hazard ratios (HRs) and 95% confidence intervals (CIs) derived from Cox proportional hazards models were used to quantify the association. Results: There was a graded increase in the incidence of type 2 diabetes among individuals with increasing levels of SUA. In the whole study cohort, compared to quartile 1, the multivariable-adjusted HRs (95% CIs) of type 2 diabetes in quartile 2, quartile 3, and quartile 4 were 1.69 (0.76–3.76), 1.86 (0.88–4.26), and 1.94 (1.05–4.05), respectively (P for trend = 0.004). This positive gradient for the risk of type 2 diabetes across quartiles of SUA was evident in both genders and across age groups. Conclusions: This study supports that high uric acid concentrations are associated with increased diabetes risk, independent of other known risk factors. These data expand on well-established associations between SUA level and metabolic syndrome, and extend the link to the future risk of type 2 diabetes.

## 1. Introduction

Type 2 diabetes is an enormous and growing clinical and public health problem and is the biggest endocrine driver for global burden of disease. According to the figures given by the NCD Risk Factor Collaboration, global age-standardized diabetes prevalence increased from 4.3% in 1980 to 9.0% in 2014 in men, and from 5.0% to 7.9% in women. On a global scale, diabetes particularly hits middle-aged people aged between 40 and 59 years, which causes serious economic implications [1]. Furthermore, type 2 diabetes has become an important public health challenge in the Chinese population living in mainland China, Taiwan, Hong Kong, and Singapore, which accounts for at least one fifth of the global population [2]. It has been suggested that diabetic epidemic will continue even if the level of obesity remains constant [1]. Thus, identifying risk factors responsible for the development of type 2 diabetes is urgently required for the prevention of type 2 diabetes.

Uric acid is generated during nucleotide and adenosine triphosphate (ATP) metabolism and comprises the end product of human purine metabolism [3]. Serum uric acid (SUA) has been associated with various cardiovascular and metabolic conditions such as hypertension, obesity, heart failure, and metabolic syndrome in large population-based studies [4,5,6]. In addition, epidemiological data suggest a positive association between SUA level and fasting plasma glucose and impaired fasting glucose [7,8]. Biologically, uric acid plays an important role in worsening of insulin resistance in animal models by inhibiting the bioavailability of nitric oxide, which is essential for insulin-stimulated glucose uptake [9,10]. It has been hypothesized that hyperuricemia might be a risk factor for the development of type 2 diabetes, but the casual association between hyperuricemia and type 2 diabetes remains controversial. Since elevated SUA levels are often associated with established type 2 diabetes risk factors, such as alcohol consumption and metabolic syndrome, it is still unclear whether SUA is merely a risk marker or an independent risk factor for diabetes [11]. Little prospective data on the topic are available, particularly in the general population. Indeed, studies of individuals with impaired glucose levels have suggested that hyperuricemia is an independent risk factor for diabetes [8,12]. Furthermore, the Rotterdam study of individuals 55 years and older reported similar results [13]. These findings call for confirmation by prospective, general-population-based data. To address this issue, we examined the independent association between SUA levels and the future risk of type 2 diabetes in a population-based cohort of Taiwanese adults.

## 2. Materials and Methods

### 2.1. Study Design and Population

Data for this study came from the Taiwanese Survey on Hypertension, Hyperglycemia, and Hyperlipidemia (TwSHHH) that was conducted in 2002. Participants in the TwSHHH were drawn from a subsample of the National Health Interview Survey (NHIS) conducted in 2001. Individuals selected for the NHIS were based on a multistage stratified systematic sampling scheme, with the method of probability proportional to size. Overall, 6592 households (26,658 individuals) were sampled from the entire Taiwan area. Because implementing a biomarker screening for all NHIS participants was not affordable, one half of the NHIS chosen townships/districts were randomly selected for the TwSHHH. In total, 3296 households with 10,292 individuals were randomly selected for the TwSHHH. Of these 10,292 subjects, 7578 (73.6%) completed interviews and 6600 (64.1%) permitted blood pressure and other biomarker measurements. A subsequent follow-up program, named TwSHHH-II, was initiated in 2007. Those 6600 individuals with complete data collection in the TwSHHH were enrolled in the TwSHHH-II. Subjects were excluded if they refused to participate (n = 1095), were dead (n = 242), and had inability to make contact (n = 581). Accordingly, there were 4682 individuals including in the TwSHHH-II, resulting in a response rate of 70.9%. Differences in sex and age distributions were not statistically significant between those participating the TwSHHH-II and non-participants. For study purposes, study subjects with a baseline diagnosis of type 2 diabetes on the basis of fasting glucose ≧ 126 mg/dL or the use of hypoglycemic agents (n = 299), those with missing data on SUA (n = 3), those who were taking uric acid-lowering medications (n = 99), and subjects without data on blood pressure, body mass index (BMI), waist circumference, serum lipid profiles, or fasting plasma glucose (FPG) measurements at baseline recruitment (n = 151) were excluded. The final analytic sample included 4130 participants (mean (±SD) age, 41.7 ± 15.9 years; 46.1% males). The protocols for the TwSHHH and TwSHHH-II were approved by the Institutional Review Board at the Bureau of Health Promotion, Department of Health, Executive Yuan in Taiwan. Written informed consent was obtained from all participants in the TwSHHH and TwSHHH-II.

### 2.2. Data Collection and Measurements

At baseline recruitment, participants received questionnaire interviews and several measurements, including anthropometry and blood pressures, were taken by well-trained nurses under a standardized protocol. Anthropometric measurements, including body weight, height, and waist circumference, were performed for each participant. Accordingly, BMI was determined as weight (in kg) divided by height (in m^2^). In addition, systolic blood pressure (SBP) and diastolic blood pressure (DBP) were measured two times from each subject by well-trained nurses using a random-zero mercury column sphygmomanometer in the TwSHHH, and an electric sphygmomanometer (BP3AC1-1, Microlife Cooperation, Berneck, Switzerland) in the TwSHHH-II. A third BP measurement was made if the first two blood pressure readings differed by more than 10 mm Hg. Then, the average of the two closest readings was calculated to determine the reported BP for each participant. Furthermore, a blood sample was collected for each study subject after a 12-hour overnight fast. In the present study, SUA was measured using the colorimetric uricase-peroxidase system. Additionally, biochemical variables, including total cholesterol, triglycerides, and FPG were determined with an automatic analyzer (Vitros 750, Orthoclinical Diagnostics Inc., Johnson & Johnson, New Brunswick, NJ, USA). For additional lipid profiles, electrophoresis was performed to measure high-density lipoprotein cholesterol (HDL-C) and low-density lipoprotein cholesterol (LDL-C) (Helena Rep; Helena Laboratories, Beaumont, TX, USA). All measurements were taken with blind quality control specimens in the central laboratory.

### 2.3. Statistical Analysis

We classified participants on the basis of sex-specific quartiles of SUA. The quartiles of SUA were 6.2, 7.2, 8.2, and >8.2 mg/dL in men, and 4.5, 5.3, 6.3, and >6.3 mg/dL in women. For descriptive analyses across the quartile group of SUA, we used chi-square analyses for categorical variables and ANOVA for continuous traits. In this study, the primary outcome measure was incident type 2 diabetes (defined as FPG ≧ 126 mg/dL or use of hypoglycemic medications). Multivariable Cox proportional hazards models were used to estimate the hazard ratios (HRs) and 95% confidence intervals (CIs) for the relationship between baseline SUA and incident type 2 diabetes, after adjusting for potential confounders. The potential confounders included in the model were age, sex, cigarette smoking, alcohol drinking, regular exercise level, BMI, waist circumference, SBP, and levels of glucose and low-density lipoprotein cholesterol. The proportional hazards assumption was fulfilled for all factors used in the Cox model, shown by parallel lines of –log[–log(survival)] versus log of follow-up time [14]. It has been advocated that the association between SUA and glucose-related endpoints was especially pronounced among women [15,16]. Further, the population is characterized by different lifestyles such as habits of smoking, alcohol drinking, exercise, and differences in lipid profiles according to both gender and age (data not shown). Thus, sensitivity analyses were applied to evaluate the consistency of the association between SUA levels and diabetes risk on the basis of stratified analyses by age and sex (men with age less than 50 years, men with age greater than or equal to 50 years, women with age less than 50 years, and women with age greater than or equal to 50 years). All the analyses were performed using SAS version 9.2 (SAS Institute, Cary, NC, USA), and all the statistical tests were 2-tailed with an α level of 0.05.

## 3. Results

The study cohort comprised 4130 individuals with mean age 41.7 ± 15.9 years; 2227 (53.9%) were women; average BMI and waist circumference were 23.4 ± 3.7 kg/m2 and 79.1 ± 11.1 cm, respectively. The mean follow-up time was 5.4 years and comprised 22,247.7 person-years. A total of 116 new cases of type 2 diabetes were recorded during 2002–2007, resulting in an incidence rate of type 2 diabetes of 5.21 per 1000 person-years.

Table 1 presents the baseline characteristics of study participants by quartiles of SUA level. Individuals in the higher uric acid quartiles were more likely to be older. In addition, higher levels of SUA were significantly positively associated with BMI, waist circumference, blood pressure, FPG, and serum levels of total cholesterol, LDL-C, and triglycerides. However, elevated levels of SUA were significantly negatively related to serum level of HDL-C.

Table 2 shows the HRs and 95% CIs for type 2 diabetes, according to uric acid quartile at baseline. In the whole study cohort, compared to the lowest quartile, the multivariable-adjusted HRs (95% CI) of type 2 diabetes associated with second quartile, third quartile, and the highest quartile of SUA were 1.69 (0.76–3.76), 1.94 (1.02–8.26), and 2.04 (1.04–5.05), respectively (P for trend <0.001). This positive gradient for the risk of type 2 diabetes across quartiles of SUA was apparent in both genders and across age groups (Table 3).

## 4. Discussion

In this prospective study of a nationally representative sample of Taiwanese adults, we found higher levels of serum uric acid were associated with an increased risk of developing type 2 diabetes. This positive association persisted in both genders and was independent of other known risk factors of type 2 diabetes, including age, sex, cigarette smoking, alcohol drinking, regular exercise level, BMI, waist circumference, SBP, and levels of glucose and low-density lipoprotein cholesterol. Overall, these findings provide prospective evidence that individuals with higher serum uric acid are at an increased future risk of type 2 diabetes, independent of other known risk factors.

Our data provide prospective confirmation of previous findings on the relationship between SUA levels and the risk of type 2 diabetes [12,13,17,18,19,20,21,22,23,24,25,26]. The Rotterdam study showed that the risk of developing type 2 diabetes in the top quartile of uric acid was 1.68 times that in the lowest quartile [13]. Prospective data from two generations of the Framingham Heart Study provide evidence that individuals with high SUA are at a higher future risk of type 2 diabetes, independent of other known risk factors [19]. Furthermore, a case-cohort nested in the European Prospective Investigation into Cancer and Nutrition-Netherlands Study supports that high SUA concentrations are associated with increased diabetes risk, independent of other risk factors [21]. Indeed, several meta-analyses provided strong evidence that a high level of SUA is independent of other established risk factors for developing type 2 diabetes; each 1mg/dl increase in SUA resulted in a 13%–17% increase in the risk of type 2 diabetes. Interestingly, the effect of a 1 mg/dl increment in SUA has been found to be comparable to a 1 kg/m^2^ increment in BMI [27,28,29]. In addition, The Finnish Diabetes Prevention Study found that having a SUA level within the top tertile was associated with a twofold increase in the risk of type 2 diabetes, compared with the lowest tertile among individuals with impaired glucose tolerance [8]. Furthermore, Wu et al. identified that SUA was positively associated with hyperinsulinemia and insulin resistance in prediabetic patients [30]. The Rotterdam Study agrees with the notion that SUA is more closely related to the early stages of the development of type 2 diabetes than late-phase mechanisms [23].

The biological mechanisms underlying the relationship between SUA and diabetes risk remain a matter of debate. Data from animal and human experiments have indicated that UA mainly affects diabetes through inflammation, oxidative stress, and endothelial function damage [9,26,31]. It is also possible that elevated SUA may reflect prediabetes status, and SUA might be more closely associated with early-phase pathogenic mechanisms that contribute to the development of type 2 diabetes [20,23,30,32]. Potential biological mechanisms underlying the association between SUA and diabetes risk remain to be clarified by future studies.

The main strengths of our study included its prospective nature, which minimizes the chance of reverse causation. In addition, the current study was performed in a nationally representative sample of a Taiwanese adult population; thus, the findings are likely to be generalizable to Taiwanese adults. On the other hand, the results of the present study need to be interpreted within the context of some limitations. The current study did not collect sufficient information on medication use and dietary habits to include them in data analyses. Although our analyses made adjustments for important confounders, there remains the possibility that the observed relationship was secondary to residual confounding. In addition, because baseline SUA concentrations were measured only once, our results may be prone to intraindividual variations that might have attenuated our results.

## 5. Conclusions

In conclusion, results from this nationally representative sample of Taiwanese adults provide evidence that individuals with elevated SUA levels are at a higher future risk of type 2 diabetes, independent of other known risk factors. These data expand on well-established associations between hyperuricemia and the metabolic syndrome, and extend the link to the future risk of type 2 diabetes.

## Figures and Tables

**Table 1:** Baseline characteristics of the study population by quartiles of serum uric acid.

	Quartiles of Serum Uric Acid (mg/dL)				
Variable	Quartile 1	Quartile 2	Quartile 3	Quartile 4	P Value
No. of subjects	1010	1108	997	1015	
Age (years, mean ± SD)	40.7 ± 15.1	40.8 ± 15.6	42.3 ± 16.6	43.1 ± 16.3	0.001
BMI (kg/m^2^, mean ± SD)	22.2 ± 3.0	22.9 ± 3.5	23.5 ± 3.5	25.0 ± 4.2	<0.001
WC (cm, mean ± SD)	76.3 ± 9.8	78.0 ± 11.1	79.4 ± 10.8	83.1 ± 11.6	<0.001
Blood pressure (mmHg, mean ± SD)					
Systolic	111.1 ± 15.8	112.6 ± 16.3	114.2 ± 16.7	118.8 ± 18.6	<0.001
Diastolic	72.2 ± 10.2	73.6 ± 11.0	74.3 ± 10.6	77.6 ± 11.6	<0.001
FPG (mg/dL, mean ± SD)	87.4 ± 9.2	88.3 ± 9.1	88.8 ± 9.4	90.2 ± 9.9	<0.001
Lipids (mg/dL, mean ± SD)					
Total cholesterol	174.1 ± 34.1	179.2 ± 32.8	184.4 ± 37.6	191.8 ± 36.5	<0.001
HDL-C	56.6 ± 14.4	55.8 ± 13.8	55.8 ± 15.1	54.5 ± 15.6	0.02
LDL-C	108.3 ± 24.9	113.0 ± 25.3	115.8 ± 27.1	121.7 ± 28.3	<0.001
Triglycerides	99.8 ± 53.8	111.5 ± 65.7	126.9 ± 81.5	147.6 ± 91.2	<0.001
Cigarette smoking (No., %)	209 (21.7)	233 (21.8)	179 (18.8)	200 (20.5)	0.334
Alcohol intake (No., %)	239 (23.7)	268 (24.3)	243 (24.5)	247 (24.4)	0.979
Regular exercise (No., %)	523 (52.0)	573 (51.9)	562 (56.6)	549 (54.2)	0.105

BMI = body mass index; WC = waist circumference; FPG = fasting plasma glucose; HDL-C = high-density lipoprotein cholesterol; LDL-C = low-density lipoprotein cholesterol.

**Table 2:** The association between serum uric acid levels and the risk of type 2 diabetes.

Parameters	Quartiles of Serum Uric Acid (mg/dL)				
	Quartile 1	Quartile 2	Quartile 3	Quartile 4	P_trend_
The whole cohort					
Participants, n	1010	1108	997	1015	
Person-years	5448.9	5976.5	5376.7	5445.6	
Incident diabetes, n	18	27	34	37	
Incidence rate (per 1000)	3.30	4.52	6.32	6.79	
Adjusted HR (95% CI)	1.00	1.69	1.94	2.00	<0.001
	(ref.)	(0.76–3.76)	(1.02–8.26)	(1.04–5.05)	

HR = hazard ratio; CI = confidence interval. Hazard ratios were adjusted for age, sex, cigarette smoking, alcohol drinking, regular exercise level, body mass index, waist circumference, systolic blood pressure, and levels of glucose and low-density lipoprotein cholesterol.

**Table 3:** Subgroup analyses of the association between serum uric acid levels and risk of type 2 diabetes by sex and age.

Parameters	Quartiles of Serum Uric Acid (mg/dL)				
	Quartile 1	Quartile 2	Quartile 3	Quartile 4	P_trend_
Men with age <50 years					
No. of participants	362	344	324	327	
Person-years	1958.5	1842.7	1743.6	1756.5	
No. of incident diabetes	6	6	11	13	
Incidence rate (per 1000)	3.06	3.26	6.31	7.40	
Adjusted HR ss(95% CI)	1.00 (ref.)	1.12 (0.30–2.88)	1.78 (1.28–4.09)	2.69 (1.08–6.71)	<0.001
Men with age ≧ 50 years					
No. of participants	141	146	127	132	
Person-years	756.3	779.0	675.3	703.1	
No. of incident diabetes	3	4	3	8	
Incidence rate (per 1000)	3.97	5.13	4.44	11.38	
Adjusted HR (95% CI)	1.00 (ref.)	1.39 (1.15–2.94)	1.22 (1.02–4.16)	2.78 (1.14–6.76)	0.040
Women with age <50 years					
No. of participants	430	384	414	366	
Person-years	2305.6	2077.8	2232.5	1967.0	
No. of incident diabetes	5	6	7	10	
Incidence rate (per 1000)	2.17	2.89	3.14	5.08	
Adjusted HR (95% CI)	1.00 (ref.)	1.12 (0.34–3.69)	1.42 (1.21–4.82)	2.24 (1.54–5.71)	<0.001
Women with age ≧ 50 years No. of participants	164	163	157	149	
Person-years	885.8	872.5	832.8	760.7	
No. of incident diabetes	7	7	8	12	
Incidence rate (per 1000)	7.90	8.02	9.61	15.77	
Adjusted HR (95% CI)	1.00 (ref.)	1.13 (0.40–3.42)	1.24 (1.02–4.18)	2.18 (1.26–5.55)	0.002

HR = hazard ratio; CI = confidence interval. Hazard ratios were adjusted for cigarette smoking, alcohol drinking, regular exercise level, waist circumference, systolic blood pressure, and levels of glucose and low-density lipoprotein cholesterol.
